# Highly Efficient Deep Learning-Enabled Parameterization and 3D Reconstruction of Traditional Chinese Roof Structures

**DOI:** 10.3390/s26031054

**Published:** 2026-02-05

**Authors:** Ruisi Ou, Fan Yang, Lili Li, Liyu Cheng, Lile Qian, Ye He, Mingliang Che, Chi Zhang

**Affiliations:** 1 Nantong Key Laboratory of Spatial Information Technology R&D and Application, College of Geographic Science, Nantong University, Nantong 226019, China; 2321110065@stmail.ntu.edu.cn (R.O.); 2421320001@stmail.ntu.edu.cn (L.C.); 2421320003@stmail.ntu.edu.cn (L.Q.); 2521320001@stmail.ntu.edu.cn (Y.H.); dawnche@ntu.edu.cn (M.C.); zhangchi1982@ntu.edu.cn (C.Z.); 2 Jiangsu Yangtze River Economic Belt Research Institute, Nantong University, Nantong 226019, China; 3 Nan Tong Surveying & Mapping Institute Co., Ltd., Nantong 226019, China

**Keywords:** symmetry detection, traditional Chinese roof, 3D point cloud, deep learning, HBIM

## Abstract

Ancient Chinese architecture, with its typical symmetrical structures, curved roofs, and upturned eaves presenting a unique architectural aesthetic, is a treasure of Chinese culture. Recently, unmanned aerial vehicle oblique photogrammetry and laser scanning technology have greatly facilitated the realistic replication of ancient buildings and have become crucial data sources for the HBIM of ancient buildings. However, parameter extraction and geometric model representation are more difficult because of the curved surfaces and upturned eaves of traditional Chinese roofs. As symmetrical features are typical of ancient Chinese architecture, the parameter quantity and modelling difficulty of the model representation can be effectively reduced by recognizing the symmetrical structure of traditional Chinese roofs and using “mirror replication” to quickly generate the other half of the model. Accurate symmetry detection and highly efficient parameter extraction are crucial for the HBIM of traditional Chinese roofs. Therefore, in this study, a deep learning network, namely, TCRSym-Net, is proposed to identify the symmetry from point clouds of traditional Chinese roofs. Each roof point cloud is then relocated and reoriented to obtain longitudinal and cross sections, and parametric modelling scripts are coded in Dynamo to model traditional Chinese roofs via curve lofting and solid Boolean operations. The experimental results reveal that the symmetry detection network is effective for symmetry detection, and five different types of traditional Chinese roofs are successfully recreated, which confirms the dependability of the method.

## 1. Introduction

As a carrier of Chinese civilization and art, ancient Chinese architecture has become a symbol of Eastern aesthetics because of its unique artistic form and profound cultural connotations. The main material that was used in ancient Chinese architecture was wood. Owing to the fragility of wooden structures and natural or man-made disasters, many ancient Chinese buildings have disappeared into the long river of history [1]. Therefore, 3D digital modelling technology is becoming increasingly necessary for understanding and repairing these buildings. With the rapid updating and development of cities and the increasing risk of irreversible damage to historical buildings, integrating the cultural heritage of ancient architecture with digital archives has become an inevitable trend [2].

Traditional surveying and recording methods are not sufficient to satisfy the demands of highly efficient modelling of historical buildings, and parameterizing nongeometric attributes, such as historical information and material data, is difficult [3,4]. With its advantages in parametric modelling and information integration, historical building information modelling (HBIM) technology has emerged as an essential tool for the preservation of ancient architectures [5,6].

Early studies focused primarily on constructing modular systems [7,8] by following the regulations and principles of the *cai-fen* system to construct BIM components, and parametrically modelling ancient architectural structures through key control parameters. These approaches meet the requirements of most standardized ancient architectural models and are typically used for model construction during the design phase. However, constructing systems of this type on the basis of rules requires the effective acquisition of control parameters, and traditional tape measures and total stations require surveyors to possess professional knowledge of ancient architecture. The emergence of laser scanning and oblique photogrammetry technologies has effectively improved the efficiency of field data collection, and these methods are being increasingly applied in the field of heritage building protection [9,10]. However, efficiently obtaining parameters and quickly constructing BIM models on the basis of these parameters are problems that urgently need to be solved [11].

The structures of ancient Chinese architecture can be roughly classified into three parts: foundation, body, and roof. Creating an accurate roof model is an important step in the 3D modelling process. Common types of ancient building roofs include hipped roofs, gable and hip roofs, flush/overhanging gable roofs, and four-corner tents. The roofs of ancient Chinese buildings usually have symmetrical structures, and a curved roof shape in combination with different cornice warping angles forms a unique architectural style. Owing to the curved surface and upturned eaves of traditional Chinese roofs, meeting the needs of HBIM has become quite difficult for parameter extraction and geometric model representation processes. The roofs of ancient Chinese architecture usually have symmetrical structures, and symmetry aids in reducing complexity by representing an entity with less information [12]. By identifying their symmetrical structures, the number of parameters in the geometric representation of the curved roofs of ancient buildings can be effectively reduced. The other half of the model can be generated through techniques such as “mirror replication”, thereby reducing the workload of repetitive modelling.

While symmetry identification using images has become increasingly popular, symmetry detection in ancient Chinese architecture using point clouds is still highly challenging and has undergone very little investigation [13,14]. The emergence of deep learning methods has greatly improved the ability to extract features [15,16], and many studies have used deep learning techniques to study symmetry detection methods [4]. However, there is currently a lack of relevant datasets for traditional Chinese roof modelling. Existing symmetry detection neural networks are typically designed for specific applications and struggle to meet the requirements of ancient architectural parameter extraction. Therefore, in this study, a deep learning-based symmetry detection and 3D modelling method for traditional Chinese roofs is proposed. Three aspects are among the contributions of this study:

(1) A deep neural network, namely, TCRSym-Net, is proposed for recognizing the symmetry of point clouds on traditional Chinese roofs;
(2) A local roof coordinate system is determined on the basis of symmetry information, expressions for longitudinal and cross sections and the parameters of upturned eaves of the roof are defined, and the defined roof parameters are extracted from point clouds;
(3) The curved surfaces of various traditional Chinese roof types are constructed into BIM using Dynamo modelling scripts.

The rest of this paper is structured as follows. The related works are reviewed in Section 2. The proposed symmetry detection method is described in detail in Section 3. The experiments and discussion are presented in Section 4 and Section 5, respectively. The paper is concluded in Section 6.

## 2. Related Works

As a nonrenewable carrier of cultural heritage, ancient Chinese architecture carries the historical progression of Chinese civilization and the wisdom of craftsmen. In recent years, 3D laser scanning and oblique photogrammetry technologies have been widely used to collect data from ancient architectural relics. However, this type of work is time-consuming and labour-intensive, and there is an urgent need to implement automated modelling methods. The surface modelling of roof structures, which are crucial components of ancient architecture, still faces numerous challenges, primarily because of the difficulties in extracting geometric parameters and constructing geometric models for roof surfaces. The symmetrical characteristics of ancient Chinese architecture enable the effective simplification of model parameter expressions through symmetry detection, which is crucial for high-precision geometric parameter extraction and model reconstruction. Thus, various aspects that are closely related to this study, including symmetry detection and the 3D modelling of traditional Chinese roofs, are briefly reviewed.

### 2.1. Symmetry Detection

Symmetry is characterized by visual harmony and satisfies human cognitive instincts and emotional needs. Symmetry is like the “grammar rules” of nature and defines the order of basic laws [14]. Symmetry detection is an important research topic in computer vision, and many scholars have studied symmetry detection in two-dimensional images [17]. Researchers have used the correspondences of symmetry to reconstruct shapes in various representations, such as points [18] and curves [19]. An ICP-like method proposed by Ecins et al. [18] was used for segmenting symmetric objects and retrieving their symmetries from 3D point clouds of natural settings. The method alternates between identifying correspondences between symmetric locations and fine-tuning the candidate symmetry plane based on the correspondences.

In recent years, by leveraging a vast amount of training data and deep neural networks’ expressive capabilities, significant progress has been made in 3D point cloud symmetry detection. As the first unsupervised symmetry detection method, PRS Net [20] can simultaneously identify planar reflection symmetry and rotational surface symmetry. Nevertheless, this approach has a restricted number of rotation axes and reflection planes. As the number of predicted planes and rotation axes increases, the number of network parameters increases approximately linearly [21]. Ji et al. [22] converted the problem of identifying symmetry in 3D point clouds into a problem of classifying symmetry points. This approach labels the points on the symmetry plane as positive samples. It trains a multiscale deep neural network based on the PointNet++ architecture [23]. A weighted cross-entropy loss function was employed to address the imbalance between positive and negative samples. A preliminary symmetry plane equation is computed using the RANSAC algorithm and least squares approach based on the point-by-point classification results. SymmetryNet, which was proposed by Shi et al. [24], improves symmetry detection with pointwise multitask prediction. It predicts the symmetric positions of each point and the foot points on the symmetry plane, and clusters the predicted symmetries during the inference process.

Although symmetry detection has received widespread attention, research has often focused on specific objects or datasets. Research on, and datasets for, the symmetry detection of traditional Chinese roofs are lacking, which makes meeting the needs of high-precision parameter extraction and reverse BIM for ancient Chinese buildings difficult [25]. With respect to the complex geometric shapes of ancient building roofs, traditional symmetry detection algorithms still have limitations in terms of computational efficiency and noise interference removal. Therefore, developing deep learning methods for symmetry detection in ancient buildings that meet the requirements for ancient building modelling is very important. Owing to the widespread existence of symmetry in ancient Chinese architecture, accurately and efficiently identifying the symmetry of buildings in the 3D point clouds of ancient architectural scenes is highly important for BIM object modelling.

### 2.2. Three-Dimensional Modelling of Traditional Chinese Roofs

Building model reconstruction is a vibrant and interdisciplinary research focus, drawing significant attention from the fields of photogrammetry, computer vision, and remote sensing [6]. Early research on the 3D modelling of ancient Chinese architecture focused mainly on constructing a parameterized component library of ancient buildings and generating BIM models of ancient buildings by controlling family models through key parameters [26]. Shen et al. [27] introduced a methodology for parameterizing the design and construction regulations for these roofs as outlined in the *Yingzao Fashi*. The method converts the design and construction principles for intricate curved roofs into mathematical models, allowing designers to create such architectural forms. A rule-based method was introduced by Liu et al. [8] for the creation of Song-era ancient Chinese architecture. The technique formalizes construction standards for various architectural styles and parameterizes the wooden parts of structures based on the hierarchical topology of structural patterns and the unique module system found in traditional Chinese architecture. Hu et al. [7] provided an editable initial frame and an automated level of detail (LOD) approach for modelling various styles of ancient Chinese architecture. This method can automatically simplify building models without the need for prefabricated low-poly proxies.

An increasing number of projects have used laser scanning technology or oblique photogrammetry for ancient architectural surveys [11,28] and have reconstructed models through reverse engineering modelling. The specific BIM families that have been constructed can meet the requirements for 3D modelling of ancient Chinese architecture, but require high-precision acquisition of control parameters. Extracting control parameters from point clouds or oblique photogrammetry models remains a highly complex process. This is because point clouds are scattered, unstructured data without semantic information, which makes identifying the geometric shapes of instances and extracting parameters difficult.

Segmenting each surface and replacing point clouds with geometric primitives are necessary steps in reconstructing a geometric model. When laser point clouds or oblique photogrammetry are used for data acquisition and reverse modelling of ancient Chinese architecture, two key issues need to be addressed: (1) the semantic segmentation of point clouds [29,30] and (2) primitive shape detection or geometric parameter extraction. Li et al. [1] presented a semantic classification and 3D model expression for Chinese roofs. This method involves a two-level semantic decomposition of the roof based on the characteristics of ancient Chinese-style architecture. Ji et al. [31] proposed an improved neural network, namely, DGCNN, for roof extraction, which aims to extract roof structure from point clouds automatically. Huo et al. [32] proposed a novel method for accurate 3D modelling of roof decorative components specific to Ming and Qing official-style architecture. This approach involves the establishment of a standardized template library containing a variety of decorative elements. Dong et al. [33] presented an automatic classification technique for identifying roof types in Ming and Qing Dynasty official-style architecture. The approach utilizes a hierarchical semantic network framework to extract key geometric features and analyses ridge structures through an attributed relational graph, with classification thresholds based on historical construction rules.

Roof modelling is an important component of the modelling of ancient Chinese architecture. In terms of extracting the geometric parameters of roof surfaces, existing parameter extraction methods focus mainly on regular geometric structures [8], such as planes [34] and cylinders [4]. The roof structure in traditional ancient Chinese architecture is usually a curved surface structure, which is more complex to model than a flat roof. Effectively extracting roof parameters from three-dimensional point clouds and constructing curved roof structures is difficult [35]. To meet modelling needs, researchers have used the nonuniform rational spline (NURBS) to represent curved surfaces. Barazzetti et al. [36] obtained accurate BIM models from point cloud data by reconstructing complex and irregular objects on the basis of NURBS curves and surfaces.

Traditional methods usually involve manual or semiautomatic parameter extraction, which is not convenient for the automatic modelling of traditional Chinese roofs. Thus, developing a flexible and accurate method for parameter extraction for traditional Chinese roofs with different shapes is important.

## 3. Materials and Methods

### 3.1. Overview

**Figure 1:**
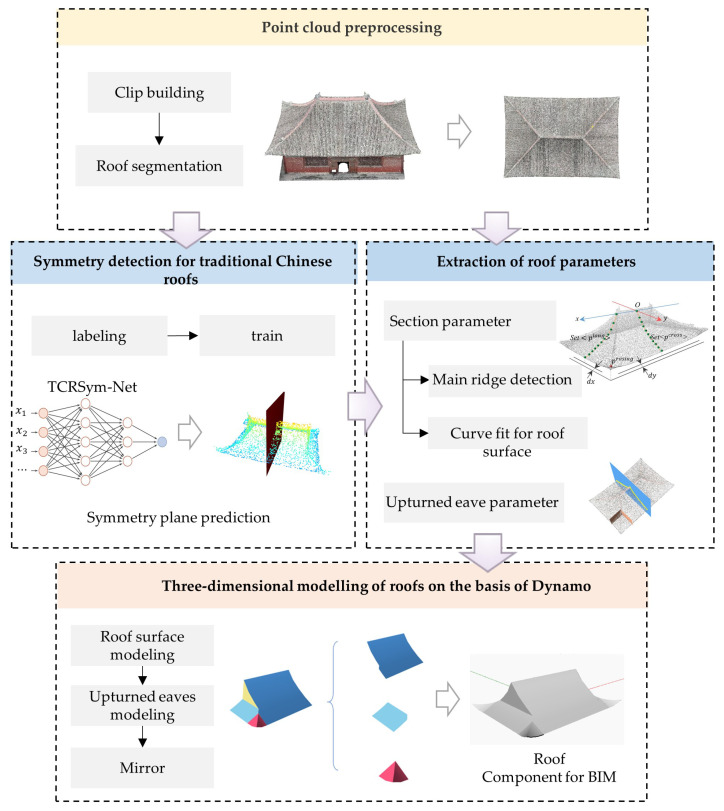
Flowchart illustrating the proposed roof reconstruction method.

In this section, we present the methodological framework for the three-dimensional (3D) reconstruction of traditional Chinese architectural roof structures. As illustrated in Figure 1, the overall workflow is systematically organized into four major phases. First, point cloud preprocessing, which includes noise filtering and roof segmentation, is performed. Second, a deep learning network, namely, TCRSym-Net, is proposed for the symmetrical detection of ancient building roofs. Third, parameter extraction and curved surface representation for roof surfaces are conducted. Finally, 3D modelling of roofs is performed using Dynamo.

### 3.2. TCRSym-Net for Symmetry Detection for Traditional Chinese Roofs

#### 3.2.1. Network Architecture

**Figure 2:**
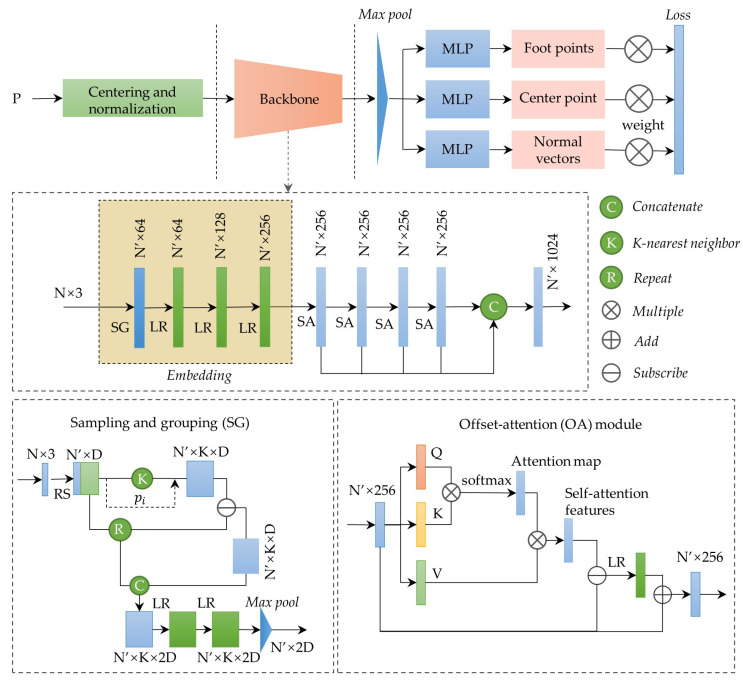
TCRSym-Net architecture.

In this study, TCRSym-Net for symmetry detection is proposed. The overall architecture of TCRSym-Net is shown in Figure 2. The input of the network is a three-dimensional roof point cloud $P=\left\{p_{i}\right\},i\in \left[1,N\right]$. The input point cloud is first centralized and normalized. The normalized data is then embedded into a new feature space. These embedded features are processed through four cascaded offset attention (OA) modules to learn discriminative pointwise features. The learned geometric features are concatenated and aggregated via a spatially weighted pooling layer to obtain a global feature representation. Finally, these features are fed into multiple task-specific prediction branches to estimate the centre point on the symmetry plane, foot points, and normal vectors.

The embedding module enhances local feature extraction capability by incorporating sampling and grouping (SG) layers and Linear-ReLU (LR) layers [12]. The SG layer first uses the farthest point sampling (FPS) algorithm to downsample the input point cloud, and uses the k-NN algorithm to search for the nearest neighbours of each sampling point; Then, it calculates the difference between the features of neighbouring points and the features of sampling points by point-wise subtraction. The difference is concatenated with the sampling points and input into the LR layers; Finally, the local features of the point cloud are obtained through max pooling.

Following PCT [37], the offset attention (OA) module uses an offset-attention mechanism to generate optimized attention features on the basis of contextual information. It first calculates the self-attention (SA) features from input features, then calculates the offset between the SA features and input features. The offset feeds the LR network and concatenates with input to ultimately obtain the OA features.

Multitask prediction branch networks contain three branches that predict the centre points on the symmetry plane, foot points, and normal vectors. Each branch contains four MLP layers and is implemented using Conv1d convolution and the ReLU function [38] to output the final predictions. All branches share an underlying feature extraction network, and each branch updates the parameters independently.

#### 3.2.2. Loss Function

For the input roof point cloud P, the symmetry plane background value is expressed as ${\hat{s}}^{ref}=\{{\hat{c}}^{s},{\hat{n}}^{s}\}$, where ${\hat{c}}^{s}$ denotes an arbitrary point on the symmetry plane, and ${\hat{n}}^{s}$ represents the corresponding normal vector. The TCRSym-Net network predicts the set of foot points $O^{s}=\{o_{i}^{s}\},i\in \left[1,N\right]$ for the input point cloud P. Each point $p_{i}\in P$ has a perpendicular foot point $o_{i}^{s}$ on the reference symmetry plane $s^{ref}$. Specifically, $o_{i}^{s}$ is the orthogonal projection of point $p_{i}$ onto the symmetry plane. Subsequently, the background value of $o_{i}^{s}$ is derived from this projection relationship, ${\hat{o}}_{i}^{s}=p_{i}-{\hat{n}}^{s}((p_{i}-{\hat{c}}^{s})\cdot {\hat{n}}^{s})$.

A composite loss function model that combines geometric alignment and symmetry constraints is used, and the multibranch prediction model predicts the normal vector $Q^{s}=\{n_{i}^{s}\},i\in \left[1,N\right]$ of the symmetry plane, any point $C^{s}=\{c_{i}^{s}\},i\in \left[1,N\right]$ on the plane, and the foot point $O^{s}$ for the input point cloud P. For each point $p_{i}$, the total loss calculation function for symmetry prediction is as follows:

(1) $$L_{i}=L_{i}^{foot}+L_{i}^{c}+w\cdot L_{i}^{conf}$$

The loss of the entire network $L_{i}$ is the sum of the branch network losses. The loss function that corresponds to each branch includes the symmetry plane point loss $L_{i}^{c}$, foot point loss $L_{i}^{foot}$ and confidence loss $L_{i}^{conf}$:

(2) $$L_{i}^{foot}=d^{2}\left(o_{i}^{s},{\hat{o}}_{i}^{s}\right)$$

(3) $$L_{i}^{c}=d^{2}\left(c_{i}^{s},{\hat{c}}^{s}\right)$$

(4) $$L_{i}^{conf}=\frac{1}{N}\sum_{j}^{N}\;CE(p_{ij},{\hat{p}}_{ij})+d^{2}(n_{i}^{s},{\hat{n}}^{s})$$

The average Euclidean distances between the predicted points and the target point are calculated for $L_{i}^{c}$ and $L_{i}^{foot}$, and the confidence loss is obtained by summing the symmetric point $CE(p_{ij},{\hat{p}}_{ij})$ and normal vector loss $d^{2}(n_{i}^{s},{\hat{n}}^{s})$. $CE(p_{ij},{\hat{p}}_{ij})$ is the cross entropy loss and $d^{2}(n_{i}^{s},{\hat{n}}^{s})$ is the mean angle error between the predicted normal and ground truths. The default value of the weight w is 0.5. During the prediction process, the DBSCAN algorithm [24] is used to cluster pointwise-estimated normal vectors, with the cluster centre vectors serving as the final predicted normal vectors. This method enables the identification of multiple symmetry planes.

### 3.3. Extraction of Roof Parameters

In previous studies, researchers have parameterized the design and construction rules that are specified in *Yingzao Fashi* for these roofs and transformed the complex curved roof design and construction rules into mathematical models to model curved roofs. In the research by Shen et al. [27], the correlation between the roof rise and the positioning of the front and rear purlins was fundamentally governed by the architectural grade of the structure. Furthermore, they determined two key parameters: the total step coefficient m and the roof curvature. These calculations were directly derived from the length of each individual rafter span, defined as the distance between adjacent structural seams. The rising and bending rules reduce the height between the rafters at different positions on the roof to form a beautiful and functional roof surface. The core of this approach lies in the calculation of the height of each important node on the roof to achieve a natural transition of the roof curve from the ridge to the eaves.

However, this method struggles to meet the demand for rapid roof surface modelling from drone scanning data, as beam span parameters are difficult to extract from top-view scans. Additionally, many historic buildings feature more flexible designs and do not satisfy traditional regulatory constraints. Therefore, building upon the research by Shen et al. [27], in this study, the use of roof point curve fitting in reverse modelling to obtain roof parameters is proposed. In the process of large-scale ancient architectural scanning and rapid modelling with drones, this method is the most straightforward and flexible.

#### 3.3.1. Definitions of the Local Coordinate System and Roof Parameters

In this study, five different roof types of traditional ancient Chinese buildings, namely, hipped roofs, gable and hip roofs, flush/overhanging gable roofs, four-corner tents, and double-eave gable and hip roofs, are determined to meet the parameter types for parametric modelling.

**Figure 3:**
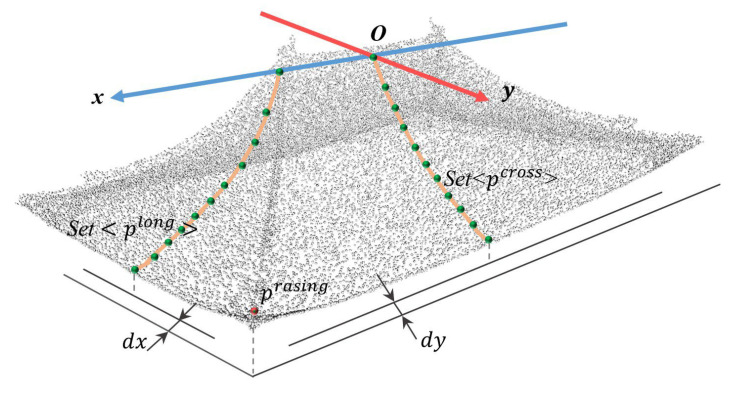
Defined local coordinate system for roof parameter description.

A local coordinate system is established to parameterize the roof geometry. The origin is set at the midpoint of the main ridge, with the *x*-axis aligned along the ridge direction and the *z*-axis oriented vertically upward. The longitudinal section is defined by a plane through the origin perpendicular to the *y*-axis, while the cross section is defined by a plane through the origin perpendicular to the *x*-axis. The geometric shape of the roof is described using parameters that are extracted from the two sections. The geometric shape of the roof observed from the front and side is shown in Figure 3.

**Figure 4:**
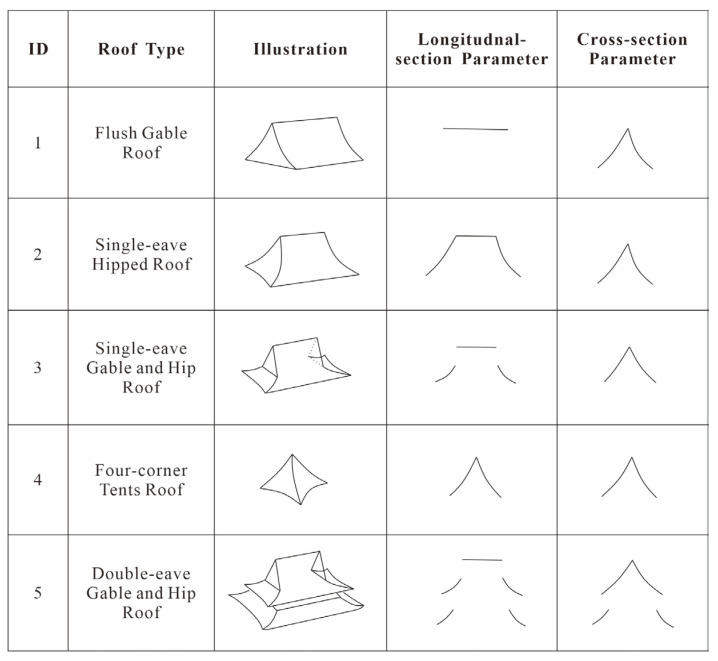
Roof types and parameter illustrations for the longitudinal section and cross section.

For the flush/overhanging gable roof, the longitudinal section contains the main ridge, the cross section contains the front slope–back slope curve, and the curve is stored via the fitted coordinate points. For the hipped roof, the longitudinal section shows the main ridge and two symmetrical slope curves, whereas the cross section shows the front slope and back slope curves. For the gable and hip roof, the longitudinal section shows the main ridge and two symmetrical slope curves, whereas the cross section shows the front slope and back slope curves. For the four-corner-tent roof, the longitudinal section shows two symmetrical slope curves, and the cross section shows the front slope and back slope curves. For the double-eave gable and hip roof, the longitudinal section shows the main ridge and two symmetrical slope curves for the upper eave, as well as two slope curves for the lower eave. The cross section shows the front and rear slope curves of the upper eave and the front and rear slope curves of the lower eaves (Figure 4).

The upturned eaves are the corner parts of the eaves in traditional Chinese architecture and are named for their upwards curve resembling a bird’s wing. They primarily connect the adjacent sloping eaves of the roof. To effectively construct a three-dimensional model of the architectural upturned eaves, the parameters that are recorded for each upturned eave include the following: the rising point, the offset distance *dx* from the starting point of the eave in the longitudinal section and the offset distance *dy* from the starting point of the eave in the cross section.

#### 3.3.2. Roof Parameter Extraction Method

**Figure 5:**
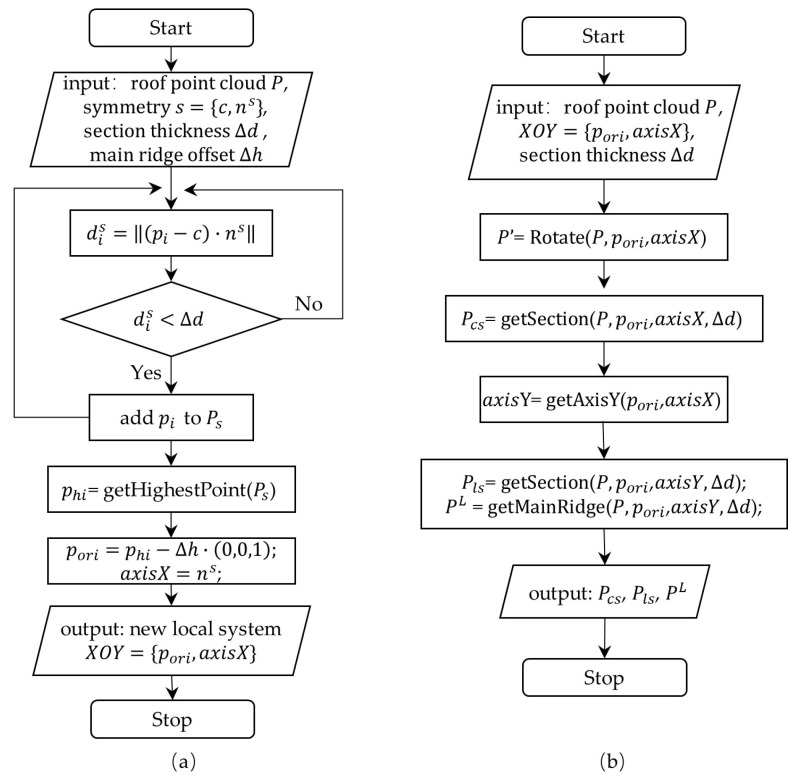
Relocation algorithm and longitudinal and cross section acquisition algorithm. (**a**) Algorithm to relocate of the local coordinate system; (**b**) algorithm to acquire longitudinal section and cross section.

**Figure 6:**
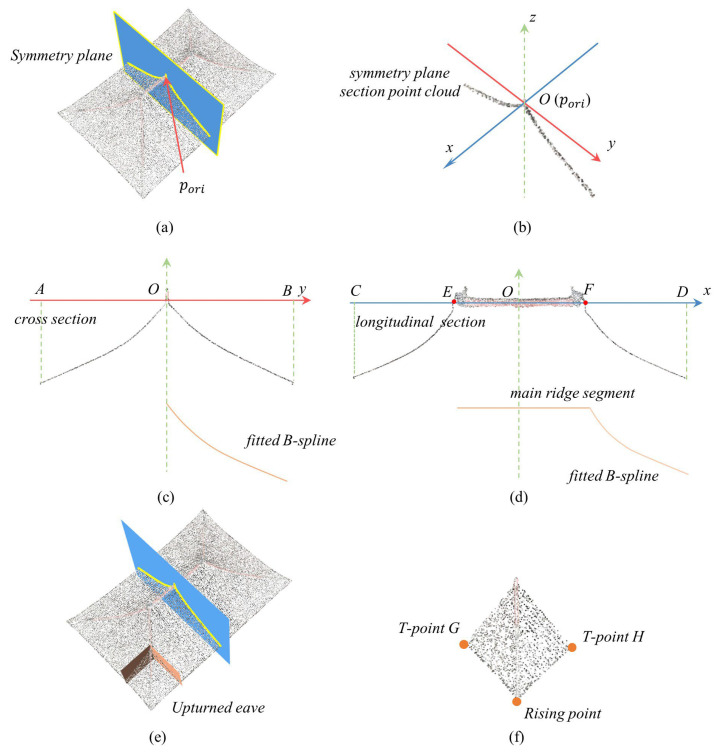
Roof parameter extraction. (**a**) Symmetry plane, (**b**) relocation of the local coordinate system, (**c**) curve fitting in the cross section, (**d**) main ridge and curve fitting in the longitudinal section, (**e**) determination of the upturned eave region, and (**f**) rising point and T-points that are extracted for the corner of the upturned eave.

To create a roof model with defined roof parameters, these parameters should be extracted from the point clouds. The parameters that are extracted for a curvilinear roof are of two main types: roof surface parameters and upturned eave parameters. After symmetry detection via the TCRSym-Net neural network, a symmetry plane $s=\{c,n^{s}\}$ perpendicular to the main ridge is chosen to segment the symmetry plane section point cloud. The distance from a point $p_{i}$ to the symmetry plane is $d_{i}^{s}=\left\|\left(p_{i}-c\right)\cdot n^{s}\right\|,\left\|n^{s}\right\|=1$, and points where $d_{i}^{s}<∆d$ are used to form the symmetry plane section point cloud (Figure 5a). The highest point $p_{hi}$ on the symmetry plane section point cloud is selected as the origin point of the coordinate system (Figure 6a,b). For ridges with offsets, the offset value ∆h is manually set. $p_{ori}$ is transformed to a new point $p_{ori}=p_{hi}-∆h\cdot (0,0,1)$.

The origin point $p_{ori}$ and symmetry plane normal vector are used to perform a rotation transformation on the roof point cloud. The cloud is rotated in the east direction, which is set as the *x*-axis. Afterwards, a plane perpendicular to the *x*-axis is used to create a cross-section $P_{cs}$, and a plane perpendicular to the *y*-axis is used to create a longitudinal section $P_{ls}$ (Figure 5b).

The main ridge parameter is derived from the longitudinal section point cloud. Ridge points are identified by projecting the roof point cloud onto line L along the *x*-axis, subject to the condition $d_{i}^{L}<∆d$. Given two points $p_{C}$ and $p_{D}$ on line L, the point $p_{i}$ projected onto L is denoted by $p_{i}^{L}$.

(5) $$p_{i}^{L}\;=p_{i}+\frac{\left|\left(p_{D}-p_{C})\cdot (p_{i}-p_{C}\right)\right|}{\left|p_{D}-p_{C}\right|}\cdot (p_{D}-p_{C})$$

The projected points on the straight line along the *x*-axis form a segment that can be used as the main ridge segment, as shown in Figure 6d. The coordinates of the main ridge point are stored on the basis of the offset from point C. To obtain the smooth curves of the roof, we used B-splines to fit the points in the sections. B-splines are mathematical representations that can accurately model complex two-dimensional or three-dimensional free-form organic curves. As shown in Figure 6c,d, the curved line is fitted using B-splines from the section point cloud, and ten refitted points are saved as the roof surface parameters.

The upturned eave parameters include the rising point and offset distances dx and dy. The trend plane of the roof slope is used to segment the upturned eave portion (Figure 6e), the maximum value of the segmented point cloud is taken as the rising point, and the offset distances dx and *dy* are calculated. The offset distance *dx* is from the rising point along the x-direction to the starting point of the eave. The offset distance *dy* is measured from the rising point along the y-direction to the starting point of the eave. Regarding the overhang distance in ancient architecture, we determine two planes parallel to the *x*-axis and *y*-axis at a distance $d_{overhang}$ inward from the eaves and obtain the transition points. As shown in Figure 6f, the T-point (transition point) is denoted by *G* and T-point is denoted by *H*. The curve of the eaves is determined by the rising point, transition point, and eaves point.

### 3.4. Three-Dimensional Modelling of Roofs on the Basis of Dynamo

**Figure 7:**
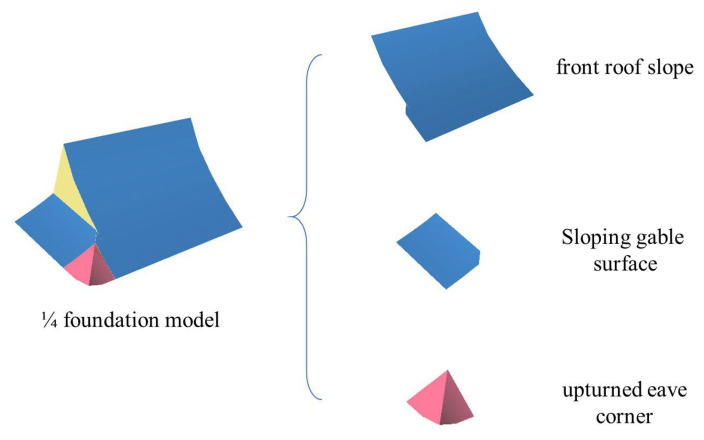
1/4 foundation modelling, in which the roof is divided into three parts: the front roof slope, sloping gable surface, and upturned eaves corners.

In this section, a parametric modelling method for traditional Chinese roofs based on Dynamo is introduced. Dynamo allows users to engage in a visual programmeming workflow in which they connect elements to establish relationships and define the sequence of actions that form custom algorithms. As a traditional Chinese roof usually has two symmetry planes, “mirror replication” technology can be easily used to quickly generate the other half of the model. In this case, a 1/4 foundation model is constructed as the base model and then mirrored to restore the overall roof model during the modelling process. For example, for the parameterized modelling of a 1/4 foundation model of a single-eave gable and hip roof, the roof is divided into four parts: the roof front slope, sloping gable surface, upturned eaves corners, and pediment surface (Figure 7).

**Figure 8:**
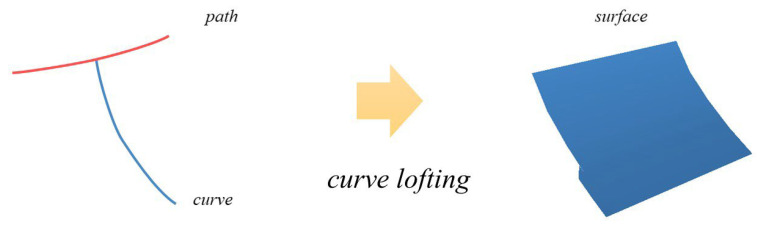
Sweeping a profile curve along a path with curve lofting.

The curve lofting method is used for roof front slope and sloping gable surface modelling. In the curve lofting method, a profile is swept along a path and is moved and aligned perpendicular to the path (Figure 8). A swept surface is defined as the surface that is generated by moving a profile curve along a trajectory curve. For a gable and hip roof, the fitted spline from the cross section $P_{cs}$ serves as the lofting curve, and the main ridge serves as the path that creates the front slope surface of the roof; the fitted B-spline from the longitudinal section $P_{ls}$ is used as the lofting curve, and the eave curve is used as the path to create the sloping gable surface.

**Figure 9:**
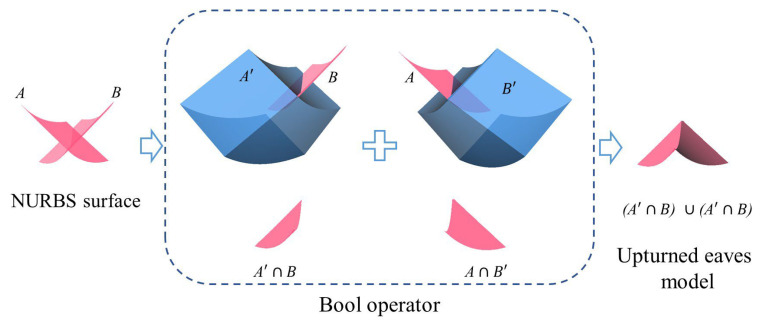
Boolean operator for creating the model of the upturned eave corner.

To model upturned eaves, we use a Boolean operation method to model the complex upturned eave surfaces (Figure 9). In this method, two adjacent roof surfaces A and B are laid out, and surface B is thickened by 5 m, whereas the other surface is thickened by 0.001 m. A and B’ are then used for solid Boolean operations to model one of the upturned eave surfaces. This process is repeated: surface A is thickened by 5 m, surface B is thickened by 0.001 m, and then the thickened surfaces A’ and B are used for Boolean operations to model another upturned eave surface. Multiple parts of the model are combined to complete the 1/4 foundation model of the roof, and the complete roof model is generated by mirroring the 1/4 foundation model twice.

The automatic BIM program was developed on the basis of Dynamo. The interpolated roof slope spline is created by applying the NurbsCurve.ByPoints method to a collection of points. Then, the roof slope spline is swept along the eave curve to create surfaces by using Surface.BySweep, and the surface is transformed into a solid with Surface.Thicken. The upturned eave models are constructed by using the Boolean difference between two solids, and the intersection points between two thickened surfaces are determined with Geometry.Interesect. Multiple parts of the model are then combined to complete the 1/4 foundation model of the roof. Finally, the complete roof model is generated by mirroring the 1/4 foundation model twice with Geometry.Mirror.

## 4. Results

### 4.1. Datasets

**Figure 10:**
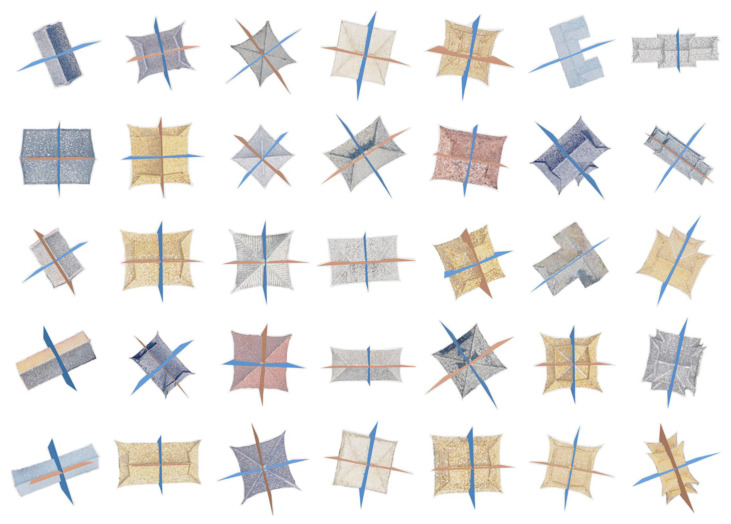
Examples from the roof symmetry detection dataset.

The experimental dataset was obtained by oblique photogrammetry and includes point cloud data for ancient buildings in areas such as Datong, Shanghai, and Nantong. The original point clouds were obtained by oblique photogrammetry [39], the obtained ground resolution ranges from 2 cm to 10 cm. The noise level is related to the accuracy of the 3D reconstruction model. The point cloud density ranges from 43.8 points/m^3^ to 21,413.6 points/m^3^. As shown in Figure 10, the roof styles include a flush gable roof, a hip roof, a single-eave gable and hip roof, a four-corner tent roof, and a double-eave gable and hip roof. The dataset contains approximately 330 unique roofs. The number of roofs of each type is given in Table 1, including 100 flush gable roofs, 54 hipped roofs, 100 single-eave gable and hip roofs, 28 four-corner tents, and 48 double-eave gable and hip roofs and others. During the preprocessing phase, we uniformly sampled 40,960 points for each roof.

Because the acquired data are vertically oriented, annotation was performed in the projected image. The original point cloud of the ancient building was segmented to obtain the roof point cloud. Afterwards, the roof point cloud was projected onto the XOY plane to obtain the projected image I. An annotation tool was used to draw a 2D line segment l along the symmetry axis on the image. The direction vector $\vec{D}$ of the line segment L was calculated, $\vec{D}$ was rotated around the *z*-axis by 90 degrees, and a new vector $\vec{N}$ was obtained as the normal vector of the symmetry plane.

Dataset augmentation was conducted to generate more samples on the basis of the existing training samples to learn as many features as possible and improve the generalization ability of the new model. Random rotations around the *z*-axis were performed on each point cloud to obtain 3300 augmented point clouds for training. We selected 80% of the point cloud points as the training set and reserved the remaining 20% of the point cloud points for validation. We additionally selected 10 roof point clouds as the test dataset and performed BIM modelling experiments.

### 4.2. Symmetry Detection for Roof Point Clouds

#### 4.2.1. Implementation Details

During network training, the input points were centred and normalized. We sampled 4096 points as input to the proposed network. The network was trained for 200 epochs, with a batch size b = 8. Afterwards, the stochastic gradient descent (SGD) [40] optimizer was used to train the network according to the loss described above. The initial learning rate lr was set as 0.001. The point cloud data were input into the training network to extract features, and after fusing global features, multiple branches were used to predict symmetry parameters. All experimental procedures were performed on a computing platform equipped with an Intel Core i7-10750H. The system was configured with 32 GB of RAM and an NVIDIA GeForce RTX 4070S GPU.

#### 4.2.2. Evaluation Metric

To evaluate and compare the proposed methods, we used the PR (precision–recall) curve to represent the training performance of the model in our study; these curves were generated by changing the threshold of the predicted confidence value [24]. To determine whether the predicted symmetry constituted a true-positive or false-positive result, we calculated the symmetry error on the basis of the difference between the predicted symmetry and the true symmetry. For an object with point $P=\{p_{i}\},i\in \left[1,N\right]$, the dense symmetry error between the predicted symmetry ${\hat{S}}^{ref}$ and the true symmetry $S^{ref}$ was calculated as follows:

(6) $$E^{ref}=\frac{1}{N}\sum_{i}^{N}\;\frac{{∥T^{ref}(p_{i})-{\hat{T}}^{ref}(p_{i})∥}_{2}}{\rho }$$

where $T^{ref}$ and ${\hat{T}}^{ref}$ represent the symmetric transformations of $S^{ref}$ and ${\hat{S}}^{ref}$, respectively, and ρ represents the maximum distance from the points in P to the symmetry plane ${\hat{S}}^{ref}$. A predicted symmetry plane is considered a true positive when it fulfils both $E^{ref}<∆\varepsilon$ and $1-cos\;(n^{s}\cdot {\hat{n}}^{s}/\left\|n^{s}\cdot {\hat{n}}^{s}\right\|)<1-cos\;(∆\theta )$.

#### 4.2.3. Qualitative Results

**Figure 11:**
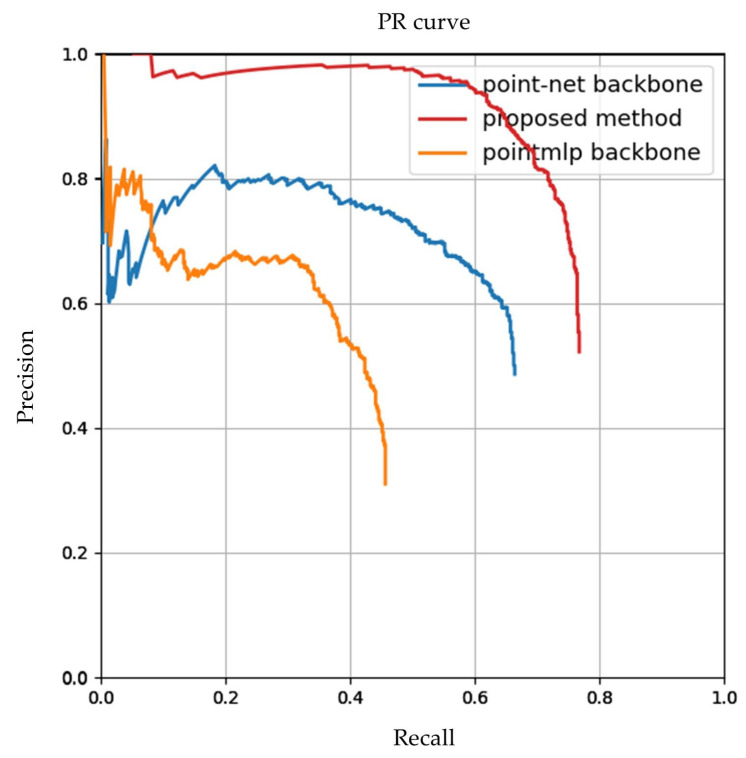
PR curves for various backbones.

As illustrated in Table 2, the proposed method achieves an AUC (Area under curve) of 0.672 and a Highest F1 score of 0.762, which is better than its counterparts built upon PointNet [41] and PointMLP [42] backbones. The PR curves for different backbones are shown in Figure 11. Since the proposed method predicts multiple normal vectors simultaneously, the prediction results may correspond to multiple ground truth values, resulting in an elevated PR curve.

In the following experiments, we applied the proposed symmetry detection method to 10 roofs in the test dataset and compared the results with those of the methods using PointNet [41] and PointMLP [42] as a backbone. The first step in the evaluation process was to match the predicted symmetry planes with the ground truth values. If the angle between the predicted normal vector and the ground truth was smaller than 5°, it was considered a correct match. Next, the angle between the predicted normal vector of the symmetry plane and the ground truth value was calculated, and the distance from the centre point on the predicted symmetry plane to the true symmetry plane was used as an evaluation metric to determine the symmetry plane detection performance of different deep learning networks on the test dataset. It can be observed that the average angle error of the method proposed in this study is 0.7563°, which is smaller than the 1.2685° of the PointNet backbone and the 1.3966° of the PointMLP backbone. The distance predicted from a centre point in the symmetry plane to the true symmetry plane by this method is 0.1043 m, which is smaller than 0.1480 m for the PointNet backbone and 0.1874 for the PointMLP backbone. The median distance error is 0.0570 m, which is also the smallest (Table 3). The evaluation results prove that the proposed method can predict symmetry parameters more accurately.

### 4.3. Roof Parameter Extraction

On the basis of the symmetry plane extracted by the TCRSym-Net neural network, the points close to the symmetry plane were selected as the section point cloud to determine the origin point of the coordinate system. The distance threshold ∆d was set to 0.05 m. The highest point on the section point cloud was set as the origin point, which was used to determine the *x*-axis. For ridges with offsets, the offset values were manually set. Afterwards, the origin point and the normal vector of the symmetry plane were used to both determine a quadrantal angle and to rotate the roof point cloud in the direction of the *x*-axis.

Next, the planes perpendicular to the *x*-axis and *y*-axis were used to construct a cross-section and a longitudinal section, respectively. The main ridge lines and ridge points were extracted from the longitudinal section. The curve line was fitted using B-splines from the section point cloud, and 10 refitted points were saved for roof surface modelling. Similarly, 10 refitted points from the side eaves were extracted at equal intervals from the cross-section.

The upturned eave parameters include the rising point and offset distances, dx and dy. The trend plane of the roof slope was used to segment the upturned eave portion, the maximum value of the segmented point cloud was taken as the rising point, and the offset distances dx and dy were calculated. The offset distance dx was measured from the rising point along the x-direction to the starting point of the eave. The offset distance dy was measured from the rising point along the y-direction to the starting point of the eave. The extracted parameters were exported as *.csv files to fulfil the 3D modelling requirements of Dynamo. The extracted parameters for the five different roofs are shown in Table 4.

### 4.4. Three-Dimensional Modelling Results

After all the roof parameters were extracted, five Dynamo scripts that were developed for traditional Chinese roof modelling were run. Two groups of roof point clouds, group A and group B, were tested in this study to verify the modelling capability of the proposed method. Each group contains five roof point clouds, including a flush gable roof, a hip roof, a single-eave gable and hip roof, a four-corner tent roof, and a double-eave gable and hip roof. All of the models were successfully created via the proposed method, as shown in Table 5 and Table 6. The index $M_{Acc}$ [43] was used to verify the accuracy of the modelled roof surfaces and is defined as follows:

(7) $$M_{Acc}=Med\left\|\pi_{j}^{T}p_{i}\right\|,\;if\;\left\|\pi_{j}^{T}p_{i}\right\|\leq r$$

where $\left\|\pi_{j}^{T}p_{i}\right\|$ measures the perpendicular distance from a vertex $p_{i}$ in the source model to a plane π in the manually created reference model. A cut-off distance r (set between 1 and 15 cm in this study) was introduced to limit the influence of incomplete or inaccurate regions in the source model. To evaluate the overall alignment between the source surfaces and the reference model, the Med function was used to calculate the median Euclidean distance from the sample points on the source surfaces to the nearest surfaces in the reference model. The accuracy values $M_{Acc}$ of the modelled roof surfaces for the test dataset are shown in Table 7.

**Figure 12:**
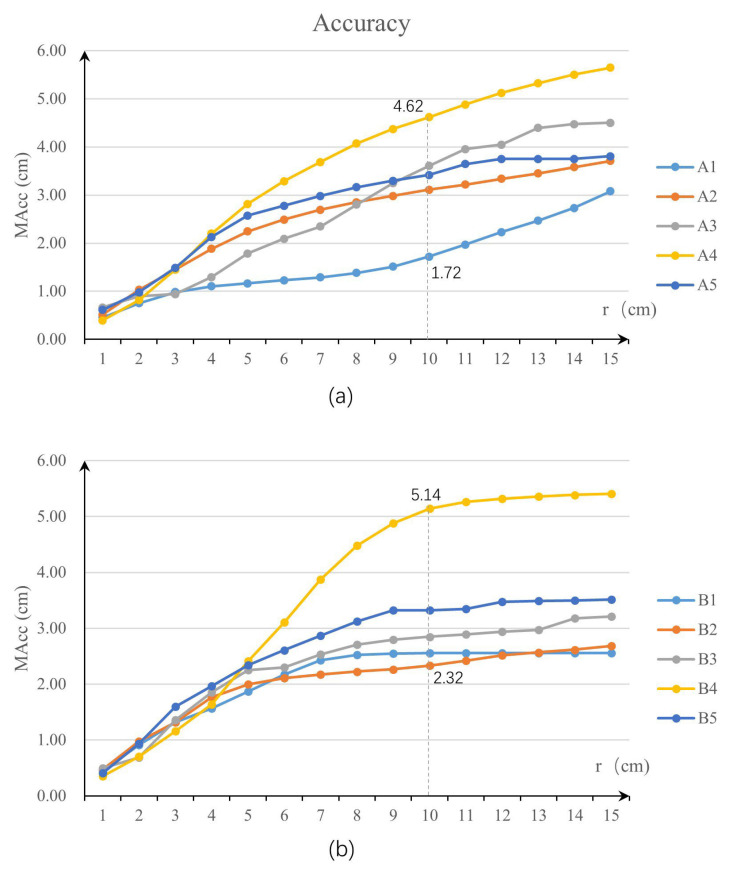
Accuracy evaluation of the roof surfaces, the dash lines indicate the $M_{Acc}$ values at 10 cm cut-off distance. (**a**) The surface accuracy for test sites in group A; (**b**) the surface accuracy for test sites in group B.

As shown in Figure 12, at a 10 cm buffer size, the surface accuracy for all test sites remained below 5 cm. Within test group A, site A1, which featured a flush gable roof, achieved the highest accuracy at 1.72 cm. In contrast, site B1, which also had a flush gable roof, resulted in a slightly lower accuracy of 2.25 cm under the same buffer conditions. The reason for this outcome is that the extracted symmetry plane for B1 is not sufficiently accurate compared with A1, as the distribution of the point cloud is uneven in B1. Furthermore, in the two experiments, the accuracy was the lowest for the four corners tents A4 and B4; this outcome may have occurred because the surface determined by the eave point, transition point, and upturned eave corner point is not sufficiently fine, and more points need to be interpolated to obtain a more accurate surface. Due to the presence of complex roof decorations, the surface accuracy of the gable and hip roof at test site A3 was lower than that of the hipped roof at test site A2. This phenomenon also occurred in the test dataset group B.

## 5. Discussion

Since symmetry detection plays a key role in roof reconstruction, in Section 4.2.2, a comparison experiment on symmetry detection is performed to verify the effectiveness of the proposed method. Compared to networks with a PointNet backbone and a PointMLP backbone, the proposed symmetry detection method achieves better symmetry detection results. The reason can be attributed to the fact that each point is well captured by the point cloud features through the offset attention mechanism, which improves the ability to extract symmetry information. The optimal assignment module and loss function proposed in this work enable it to output accurate symmetries. Supplementary Materials are available online at https://github.com/yhexie/TCRSym-Net.git (accessed on 1 February 2026).

**Figure 13:**
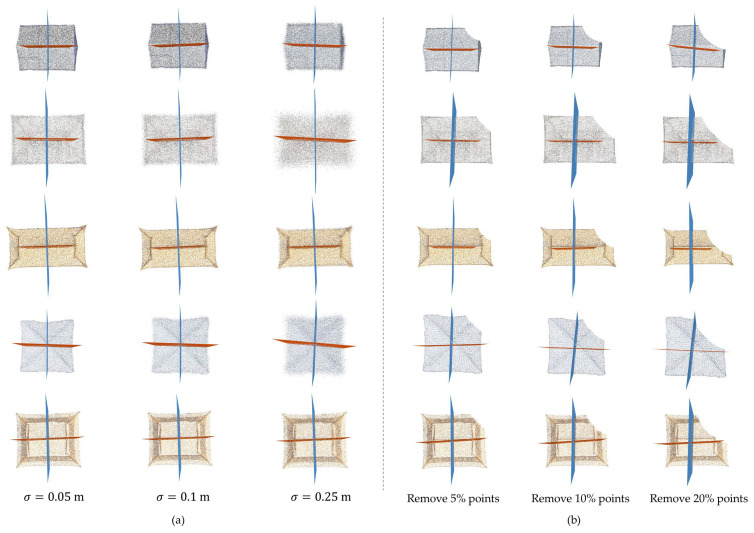
The experiments for testing the robustness of the proposed method. (**a**) Gaussian noise with standard deviations of σ = 0.05 m, 0.1 m, and 0.25 m was added to each point of the test roof point clouds; (**b**) the points were removed by 5%, 10%, and 20% of the roof point cloud.

Additional experiments were designed to test the robustness of the proposed method in the presence of noise and outliers (Figure 13a). Gaussian noise with standard deviations of σ = 0.05 m, 0.1 m, and 0.25 m was added to each point on the test roof point clouds. It is shown that the proposed method can also correctly detect symmetries, but the accuracy of detecting symmetric planes decreases with increasing noise. To test the robustness of the proposed method to incomplete point clouds, we removed the roof point clouds by a factor of 5%, 10%, and 20%. It was shown that the proposed method can still extract the symmetry information well when 5% of the point clouds are removed, but when the point cloud is removed by a factor of 10 or even 20, the extracted symmetry plane is significantly shifted, and the accuracy is significantly reduced, which does not meet the needs of modelling (Figure 13b). Therefore, the proposed method is still sensitive to missing data. When the density distribution of the point cloud is very inhomogeneous, it affects the predicted centre point of the symmetry plane and causes a significant shift.

**Figure 14:**
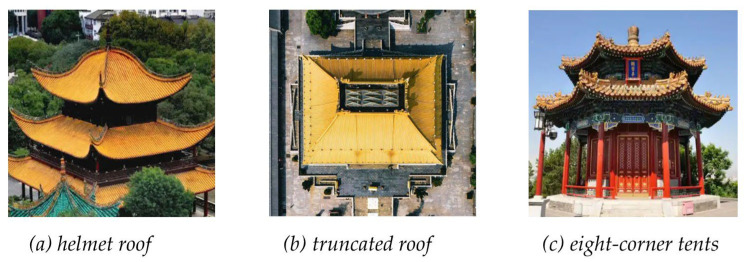
Helmet roof, truncated roof and eight-corner tents.

**Figure 15:**
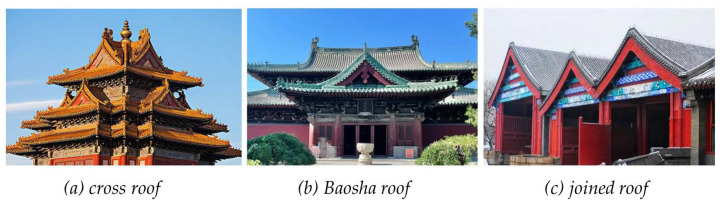
Cross roof, Baosha roof and joined roof.

The roofs that were considered in this study did not cover all types of traditional Chinese roofs, such as helmet roofs, truncated roofs and eight-corner tents (Figure 14). Thus, an expansion of the training dataset is needed. The modelling of complex ancient building structures, such as cross roofs, Baosha roofs, and joined roofs (Figure 15), has not been fully discussed. For even more complex roof types, composite roof modelling has not been achieved, which requires additional consideration in future work.

## 6. Conclusions

In this study, a deep learning network, namely, TCRSym-Net, was proposed to identify symmetry in the point clouds of traditional Chinese roofs. This network detects the symmetry plane of a roof point cloud, which is then used to relocate and reorient the roof point cloud, thus successfully determining the longitudinal and cross sections and extracting the section parameters. Roof modelling scripts were constructed by using Dynamo to construct models of various traditional Chinese roof types. The proposed method can significantly improve the automation level of BIM and reduce the cost and time investment for the digital protection of ancient buildings. Thus, it establishes a more robust digital foundation for the protection, restoration, and preservation of cultural heritage.

In the future, we will expand the existing training sample dataset and explore geometric shape identification and parameter extraction problems for complete ancient Chinese architecture modelling.

## Figures and Tables

**Table 1:** Parameters for the training data set for symmetry detection of traditional Chinese roofs.

ID	Item	Parameters
1	Number of sampling points per roof	40,960
2	Minimum size	[1.8532, 2.0453, 0.5046]
3	Maximum size	[57.9948, 6.2010, 2.6024]
4	Point cloud density range	43.8~21,413.6 points/m^3^
5	Range of diagonal lengths	2.805~63.691 m
6	Original ground resolution of drone images	2–10 cm
ID	Roof type	Number of unique roof point clouds
1	Flush gable roofs	100
2	Hipped roofs	54
3	Single-eave gable and hip roofs	100
4	Four-corner tents	28
5	Double-eave gable and hip roofs	48
Total		330

**Table 2:** Evaluation index of PR-AUC and Highest F1-score on different backbones.

Index	PointNet Backbone	PointMLP Backbone	Proposed Method
PR-AUC	0.486	0.294	0.672
Highest F1-score	0.614	0.464	0.762

**Table 3:** Evaluation of symmetry detection of the test dataset.

Background		Prediction (The Predicted Normal Vector’s Angle Error Smaller Than ∆θ=5°)						
Test Sites	Symmetry	Best Matched Prediction	Proposed Method		PointNet Backbone [41]		PointMLP Backbone [42]	
			Angle Error (Degree)	Distance Error (m)	Angle Error (Degree)	Distance Error (m)	Angle Error (Degree)	Distance Error (m)
A1	Sym1	Pred-1	0.4199	0.0403	0.6500	0.4365	1.2851	0.4355
	Sym2	Pred-2	0.1398	0.0874	0.1379	0.0038	2.0533	0.0298
B1	Sym1	Pred-1	0.2446	0.0561	1.8813	0.0295	4.2345	0.1655
	Sym2	Pred-2	1.5576	0.0954	2.5977	0.1768	3.0962	0.0091
A2	Sym1	Pred-1	0.9226	0.0033	1.7874	0.0022	2.4467	0.1344
	Sym2	Pred-2	0.1335	0.0090	2.1177	0.0174	0.4042	0.0079
B2	Sym1	Pred-1	1.1532	0.0579	0.4255	0.0828	0.3123	0.1071
	Sym2	Pred-2	0.9041	0.0180	0.6720	0.0300	0.2603	0.0231
A3	Sym1	Pred-1	0.7898	0.3393	2.0843	0.0639	0.0728	0.7751
	Sym2	Pred-2	0.8933	0.0040	1.9009	0.2447	3.7243	0.1840
B3	Sym1	Pred-1	1.1295	0.4560	1.2613	0.2825	2.7739	0.3464
	Sym2	Pred-2	0.3296	0.1226	2.1422	0.0667	0.2239	0.1111
A4	Sym1	Pred-1	0.3275	0.0625	1.0155	0.0508	0.8761	0.0318
	Sym2	Pred-2	0.8822	0.0205	2.2327	0.2931	2.7643	0.1546
B4	Sym1	Pred-1	1.2480	0.0468	0.8501	0.0121	0.0484	0.1385
	Sym2	Pred-2	1.4604	0.0405	1.0442	0.0486	0.3491	0.1072
A5	Sym1	Pred-1	0.4990	0.0334	0.0928	0.2247	0.7744	0.2128
	Sym2	Pred-2	0.9828	0.4239	0.0272	0.6557	1.0102	0.6411
B5	Sym1	Pred-1	0.1944	0.0804	1.0203	0.0756	0.3238	0.1317
	Sym2	Pred-2	0.9151	0.0884	1.4288	0.1618	0.8974	0.0013
		Mean	0.7563	0.1043	1.2685	0.1480	1.3966	0.1874
		Median	0.8877	0.0570	1.1527	0.0711	0.8867	0.1330
		Standard deviation	0.4430	0.1356	0.7920	0.1693	1.3254	0.2105

**Table 4:** Roof parameter extraction experiments.

Case	Parameters
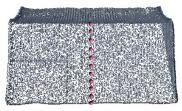	Main Axis: [−9.121803, −0.113617, 1.018555], [9.361763, −0.113617, 1.018555] Second Axis: [0.119980, −5.030745, 1.018555], [0.119980, 4.803511, 1.018555] Fitted Curve: [0.000000, −1.915039], [0.541100, −1.735352], [1.082200, −1.540039], [1.623300, −1.274414], [2.164400, −1.030273], [2.705500, −0.733398], [3.246600, −0.478516], [3.787699, −0.157715], [4.328799, 0.238770], [4.869899, 0.775879], [5.410999, 1.124023]
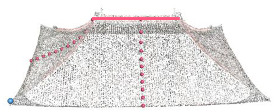	Main Axis: [−3.825644, 0.720184, 1.682293], [4.312551, 0.720184, 1.682293] Main Ridge: [2.651347, 1.682293], [5.486848, 1.682293], Fitted Curve: [0.000000, −0.562422], [0.265135, −0.412106], [0.530269, −0.267233], [0.795404, −0.108004], [1.060539, 0.058395], [1.325673, 0.233313], [1.590808, 0.429060], [1.855943, 0.627842], [2.121077, 0.847433], [2.386212, 1.156660], [2.651347, 1.682293] Second Axis: [−0.243454, −3.420081, 1.682293], [0.243454, 4.860450, 1.682293] Fitted Curve: [0.000000, −0.503857], [0.414027, −0.442457], [0.828053, −0.300898], [1.242080, −0.159391], [1.656106, −0.012772], [2.070133, 0.176414], [2.484159, 0.377151], [2.898186, 0.608349], [3.312212, 0.846260], [3.726239, 1.086050], [4.140265, 1.688667] Bounding box: [−3.825644, −3.420081, 0.000000], [4.312551, 4.860450, 0.000000] Eaves: [−3.871060, −3.436253, 0.001334], [0.045417, 0.016172]
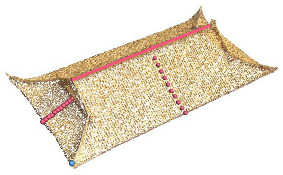	Main Axis: [−6.421559, 0.074219, 1.260220], [6.507496, 0.074219, 1.260220] Main Ridge: [1.933882, 1.260220], [10.995173, 1.260220] Fitted Curve: [0.000000, −1.254971], [0.193388, −1.148760], [0.386776, −1.090052], [0.580165, −0.985477], [0.773553, −0.886642], [0.966941, −0.800716], [1.160329, −0.683926], [1.353717, −0.619263], [1.547105, −0.521505], [1.740494, −0.437271], [1.933882, −0.353037] Second Axis: [−0.042969, −4.214520 1.260220], [0.042969, 4.362957, 1.260220] Fitted Curve: [0.000000, −1.304916], [0.428874, −1.058765], [0.857748, −0.863235], [1.286621, −0.609316], [1.715495, −0.427336], [2.144369, −0.229176], [2.573243, 0.004543], [3.002117, 0.355425], [3.430991, 0.691423], [3.859864, 1.068165], [4.288738, 1.262224] Bounding box: [−6.421559, −4.214520 0.000000], [6.507496, 4.362957, 0.000000] Eaves: [−7.169045, −4.983853, 0.525608], [0.747486, 0.769333]
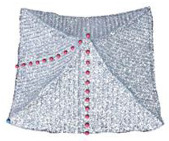	Main Axis: [−3.748123, −0.243225, 1.535645], [6.030334, −0.243225, 1.535645] Fitted Curve: [0.000000, −1.846191], [0.488923, −1.656006], [0.977846, −1.466797], [1.466769, −1.226074], [1.955692, −0.946533], [2.444614, −0.652832], [2.933537, −0.415771], [3.422460, −0.069092], [3.911383, 0.210693], [4.400306, 0.758301], [4.889229, 1.070313] Second Axis: [−1.141106, −5.823246, 1.535645], [1.141106, 5.336796, 1.535645] Fitted Curve: [0.000000, −1.688232], [0.558002, −1.688232], [1.116004, −1.501221], [1.674006, −1.214844], [2.232008, −0.945557], [2.790011, −0.662109], [3.348013, −0.403564], [3.906015, −0.104248], [4.464017, 0.204834], [5.022019, 0.504150], [5.580021, 1.065674] Bounding box: [−3.748123, −5.823246, 0.000000], [6.030334, 5.336796, 0.000000] Eaves: [−4.085910, −5.973135, 9.121094], [0.337787, 0.149889]
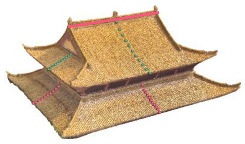	Second eave Main Axis: [−16.647789, 0.006954, 5.755520], [15.180134, 0.006954, 5.755520] Fitted Curve: [0.000000, −5.952494], [0.447442, −5.630781], [0.894884, −5.410862], [1.342326, −5.201662], [1.789768, −4.973425], [2.237210, −4.746714], [2.684652, −4.499012], [3.132094, −4.255314], [3.579536, −3.974346], [4.026978, −3.686543], [4.474420, −3.418728] Second Axis: [−0.733828, −12.860601, 5.755520], [−0.733828, 12.874510, 5.755520] Fitted Curve: [0.000000, −5.772617], [0.463336, −5.615183], [0.926671, −5.396662], [1.390007, −5.192884], [1.853342, −4.907066], [2.316678, −4.659809], [2.780014, −4.419453], [3.243349, −4.155888], [3.706685, −3.886074], [4.170021, −3.609570], [4.633356, −2.834946] Bounding box: [−16.647789, −12.860601, 0.000000], [15.180134, 12.874510, 0.000000] Eaves: [−17.279955, −13.404373, 6.180473], [0.632166, 0.543772]

**Table 5:** Experimental results of roof modelling for test dataset A.

ID	Roof Type	Pictures	Original Point Clouds	Roof Point Clouds	Roof Models
A1	Flush Gable Roof	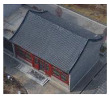	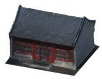	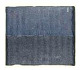	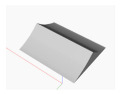
A2	Hipped Roof	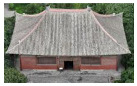	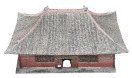	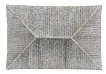	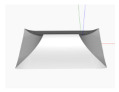
A3	Single-eave Gable and Hip Roof	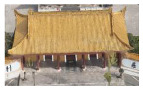	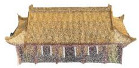	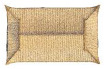	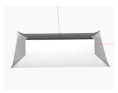
A4	Four-corner tents	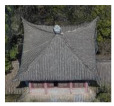	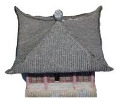	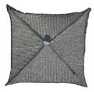	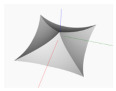
A5	Double-eave Gable and Hip Roof	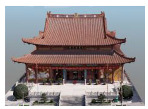	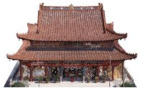	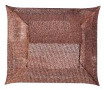	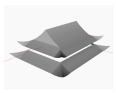

**Table 6:** Experimental results of roof modelling test dataset B.

ID	Roof Type	Pictures	Original Point Clouds	Roof Point Clouds	Roof Models
B1	Flush Gable Roof	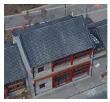	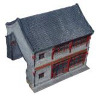	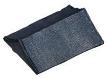	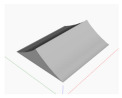
B2	Hipped Roof	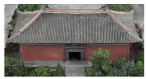	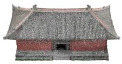	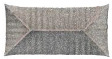	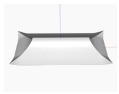
B3	Single-eave Gable and Hip Roof	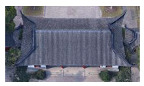	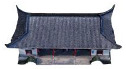	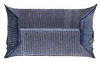	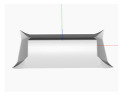
B4	Four-corner tents	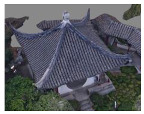	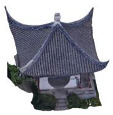	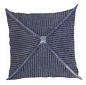	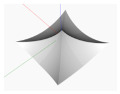
B5	Double-eave Gable and Hip Roof	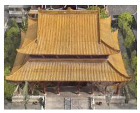	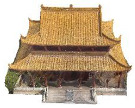	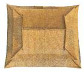	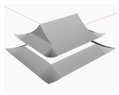

**Table 7:** The accuracy of the modelled roof surfaces for the test dataset.

Cut-Off Distance (cm)	$M_{Acc}$ (cm)									
	A1	A2	A3	A4	A5	B1	B2	B3	B4	B5
1	0.453	0.514	0.663	0.394	0.612	0.452	0.473	0.497	0.354	0.409
2	0.751	1.030	0.898	0.817	0.979	0.913	0.976	0.691	0.704	0.938
3	0.986	1.458	0.941	1.453	1.490	1.319	1.316	1.357	1.160	1.595
4	1.103	1.882	1.296	2.198	2.131	1.566	1.763	1.851	1.640	1.969
5	1.166	2.245	1.787	2.817	2.573	1.870	2.000	2.251	2.410	2.339
6	1.230	2.492	2.094	3.288	2.778	2.170	2.108	2.299	3.107	2.609
7	1.288	2.695	2.348	3.686	2.980	2.426	2.175	2.535	3.875	2.869
8	1.381	2.851	2.801	4.070	3.165	2.526	2.226	2.707	4.483	3.121
9	1.513	2.980	3.246	4.373	3.296	2.548	2.265	2.799	4.877	3.321
10	1.723	3.111	3.606	4.620	3.417	2.555	2.335	2.848	5.143	3.321
11	1.971	3.220	3.958	4.883	3.644	2.555	2.422	2.894	5.261	3.344
12	2.232	3.339	4.047	5.126	3.750	2.555	2.516	2.937	5.319	3.476
13	2.469	3.450	4.397	5.324	3.750	2.555	2.568	2.974	5.360	3.490
14	2.731	3.581	4.477	5.506	3.750	2.555	2.617	3.176	5.386	3.502
15	3.079	3.709	4.506	5.647	3.806	2.555	2.688	3.212	5.407	3.513

## Data Availability

The original contributions presented in this study are included in the article. Further inquiries can be directed to the corresponding author.

## Supplementary Materials

The source code and experimental dataset for this study are available online at https://github.com/yhexie/TCRSym-Net.git (accessed on 1 February 2026).
